# Results From 2 Cohort Studies in Central Africa Show That Clearance of *Wuchereria bancrofti* Infection After Repeated Rounds of Mass Drug Administration With Albendazole Alone Is Closely Linked to Individual Adherence

**DOI:** 10.1093/cid/ciaa1232

**Published:** 2020-08-28

**Authors:** Jérémy T Campillo, Naomi P Awaca-Uvon, Francois Missamou, Jean-Paul Tambwe, Godefroy Kuyangisa-Simuna, Gary J Weil, Frédéric Louya, Michel Boussinesq, Sébastien D S Pion, Cédric B Chesnais

**Affiliations:** 1 UMI 233, Institut de Recherche pour le Développement, Montpellier, France; 5 Université de Montpellier, Montpellier, France; 6 INSERM Unité 1175, Montpellier, France; 2 Ministère de la Santé Publique, Kinshasa, Democratic Republic of the Congo; 3 Programme National de Lutte contre l’Onchocercose, Direction de l’Epidémiologie et de la Lutte contre la Maladie, Ministère de la Santé et de la Population, Brazzaville, Republic of Congo; 4 Washington University School of Medicine, St. Louis, Missouri, USA

**Keywords:** albendazole, lymphatic filariasis, mass drug administration, treatment adherence, parametric survival analysis

## Abstract

**Background:**

Two community trials conducted from 2012 to 2018 in the Republic of Congo and the Democratic Republic of the Congo demonstrated the efficacy of semiannual mass drug administration (MDA) with albendazole (ALB) alone on lymphatic filariasis (LF). However, a high interindividual heterogeneity in the clearance of infection was observed.

**Methods:**

We analyzed trial data to assess the effect of individual adherence to ALB MDA on clearance of circulating filarial antigenemia (CFA) and microfilaremia. Community residents were offered a single dose of ALB every 6 months and tested for LF with a rapid test for CFA at baseline and then annually. CFA test results were scored on a semiquantitative scale. At each round, microfilaremia was assessed in CFA-positive individuals. All CFA-positive individuals for whom at least 1 follow-up measure was available were included in the analyses. Parametric survival models were used to assess the influence of treatment adherence on LF infection indicators.

**Results:**

Of 2658 individuals enrolled in the trials, 394 and 129 were eligible for analysis of CFA and microfilaremia clearance, respectively. After adjusting for age, sex, and initial CFA score, the predicted mean time for clearing CFA was shorter in persons who had taken 2 doses of ALB per year (3.9 years) than in persons who had taken 1 or 0 dose (4.4 and 5.3 years; *P* < .001 for both). A similar pattern was observed for microfilaremia clearance.

**Conclusions:**

These results demonstrate a clear dose-response relationship for the effect of ALB on clearance of CFA and microfilaremia.

## Teaser

Longitudinal analysis of data from 2 community trials of mass drug administration with semiannual albendazole on lymphatic filariasis (LF) demonstrated a clear dose-response treatment effect at the individual level that underlines the importance of adherence for LF elimination programs.

Lymphatic filariasis (LF) is a mosquito-borne parasitic infection caused mainly by *Wuchereria bancrofti.* The strategy for LF elimination is to interrupt the transmission cycle between humans and vectors. In African countries where onchocerciasis is endemic, programs provide annual mass drug administration (MDA) with ivermectin (IVM) plus albendazole (ALB). Bednets are also often provided to limit mosquito exposure. Treatment with IVM and ALB reduces the density of the larval stages of the parasite (microfilariae [Mf]) in the blood. However, MDA has to be repeated for many years because these drugs have a limited efficacy for killing adult worms [1]. In areas where LF is coendemic with loiasis, another filarial infection caused by *Loa loa*, this strategy is dangerous because IVM can induce serious adverse events (SAEs) in people with very high *L. loa* microfilarial densities (MFD) [2]. In these areas, alternative strategies have to be implemented. Previous clinical trials that compared the effects of various drugs on *W. bancrofti* MFD suggested that treatment with ALB alone might reduce MFD, albeit at a slower rate than after combined treatment with IVM and ALB [3–13]. ALB does not induce SAEs in patients with high *L. loa* MFD [14–16]. In 2012, the World Health Organization (WHO) proposed that MDA with ALB (preferably semiannual, and combined with integrated vector management) might be used to eliminate LF in areas where loiasis is coendemic [17]. The results of 2 community trials conducted in the Republic of Congo (Congo) and the Democratic Republic of the Congo (DRC) confirmed that this strategy was effective. At the first site, where baseline circulating filarial antigenemia (CFA) and Mf prevalences were moderate (17.3% and 5.3%, respectively) and treatment adherence was high (83%–90%), these indicators decreased to 4.7% and 0.3%, respectively, after 3 years of semiannual MDA with ALB alone [18]. In DRC, where baseline infection prevalences were higher and treatment adherence lower (56%–88%), CFA and Mf prevalences decreased from 31.6% to 8.5% and from 12.0% to 0.9%, respectively, after 4 years of semiannual MDA with ALB [19]. Although MDA with ALB alone was highly effective at the community level, considerable heterogeneity was observed in parasite clearance at the individual level; some individuals cleared their infections rapidly, while others remained infected after 8 rounds of MDA. In this study, we reanalyzed data collected from these 2 community trials to assess the effect of individual adherence to ALB treatment on the CFA and *W. bancrofti* microfilaremia clearance rates.

## METHODS

### Study Populations

The design of the 2 studies has been described elsewhere [18, 19]. In Congo, the study was conducted from 2012 to 2015 in Seke-Pembe, a village located in Mabombo Health District (Bouenza division). In DRC, the study site consisted of 2 contiguous villages (Mbunkimi and Misay) located in the Kwilu province, and the trial took place from 2014 to 2018. Study participants were tested for LF infection at baseline and then annually. Both studies were approved by ethics committees and administrative authorities in the respective countries. Adult participants signed an informed consent form. Participants aged <18 years were enrolled only after verbal assent and if 1 parent signed a consent form.

A total of 2658 individuals were examined for LF infection at least once during the 2 studies. The present analysis included all individuals who were CFA-positive at the time of their first test (which was not necessarily performed during the year when the trial started at the site) and who had at least 1 subsequent examination. Therefore, individuals who had progressed from CFA-negative to CFA-positive during the follow-up period and those who were CFA-negative at all time points tested were not included in the analysis.

### Assessment of *W. bancrofti* Infection

Annual parasitological assessments were performed for participants aged ≥5 years. LF infections were detected by CFA testing using point-of-care tests. In Congo, testing was done with the BinaxNOW Filariasis immunochromatographic card test (ICT; Alere, Scarborough, ME) in 2012, 2013, and 2014 and with the Filariasis Test Strip (FTS; Alere, Scarborough, ME) in 2015. All antigen testing in DRC was performed with FTS. ICT and FTS results were scored semiquantitatively (0, 1, 2, or 3 according to the relative intensities of the test and control lines) [18, 20]. All CFA-positive individuals were invited to return for blood sampling between 10:00 PM and 1:00 AM for assessment of *W. bancrofti* microfilaremia. MFDs were based on the arithmetic mean of the counts of two 70-µL-thick blood smears and expressed as microfilariae per milliliter.

### Drug Distribution and Assessment of Treatment Adherence

CFA-negative individuals were treated with a single tablet of ALB (400 mg) immediately after antigen testing under the direct observation of investigators. Those with positive CFA test results were treated with ALB just after collection of night blood for Mf testing. Residents who had not participated in the parasitological survey were also offered ALB treatment.

All treatments were provided under the supervision of a local healthcare worker who was also responsible for conducting a population census before each semiannual MDA. Every treatment was recorded in a drug treatment register. In addition, during the annual assessment visits, we asked the participants if they had received ALB during the previous MDA campaign, which was 6 months earlier. Therefore, for each year of the study, we could determine for each participant whether he/she had taken 2, 1, or 0 ALB tablets.

### Sociodemographics and Risk Factors for LF

At inclusion, we collected information about sex and age. At each visit using a standardized 1-page questionnaire, we also collected sociodemographic characteristics and habits that are known to be risk factors for LF, such as bednet usage and occupation (fishing, hunting, and farming), and regularly sleeping outside of the village in the bush [21, 22].

### Statistical Analyses

The events analyzed are clearance of CFA (the transition from a positive to a negative CFA test during follow-up) and clearance of microfilaremia. We used survival analysis methods [23] to account for the individual follow-up nature of the data. The start date for the survival analysis was the first visit (index date). Individual observations were censored at the end of the follow-up or at the date of the event (date of the annual parasitological survey). Each participant’s data were considered for calculation of cumulative person-years in the survival analysis.

We considered the following covariates for the analysis: sex, initial MFD (placed into 3 categories of similar sample size: 1 to 150, 150 to 300, and > 300 Mf/mL), initial CFA score (from 1 to 3), a history of fishing as an occupation (yes or no), and a history of regularly sleeping in the bush (yes or no).

We also considered the following time-varying covariates: age (categorized according to interquartile and median values: 5–17, 18–30, 31–45, and ≥46 years), number of ALB tablets taken during the previous year (0, 1, or 2), bednet use during the previous night (yes or no), and the CFA test used (ICT or FTS).

Univariate analysis of clearance rates was conducted using Mantel-Haenszel tests. Clearance rates represent the probability of occurrence of clearance in a specified period of time.

We used a parametric survival model with accelerated failure time [24] to estimate the influence of time-varying variables on infection clearance (time-to-event) [25]. Several time distributions that do not require meeting the proportional risk assumption were tested according to the Akaike information criterion (AIC). For the survival models, random effects, at both village and household levels, were assessed using results of likelihood-ratio tests. Results are presented as time ratios with 95% confidence intervals. Time ratios represent time differences to event according to the reference category. Sociodemographic data, occupation, initial infection intensity (CFA and/or MFD), and the number of ALB tablets taken each year were included in the CFA and microfilaremia clearance survival models. The type of test (ICT or FTS) was included in the CFA clearance model. The fitted models used to estimate average times to clear CFA and microfilaremia included all explanatory variables.

A mixed model with random effect at the individual level was used to describe changes in MFD according to time, treatment history, and sociodemographic information. Several transformations (linear, quadratic, first-order fractional polynomials, and second-order fractional polynomials) were tested for the time variable, and selection was made according to the AIC. As for CFA clearance analysis, random effects at village and household levels were assessed. Last, the significance of relevant interaction terms was assessed (age and sex, age and initial CFA score, age and initial MFD, age and number of ALB treatments taken, sex and initial CFA score, sex and initial MFD, sex and number of ALB treatments taken) for CFA and microfilaremia clearance and MFD change analyses. All analyses were performed using STATA v.15.1 software (StataCorp, LP, College Station, TX).

## RESULTS

### Study Participants

Of the 2658 participants enrolled in the studies, 879 were tested only once; 22 who were CFA-negative at baseline acquired CFA (15 in DRC and 7 in Congo), and 1363 were CFA-negative at baseline and all follow-up times. Thus, observations from 394 participants were available for analysis of CFA clearance for a total of 1369 person-years of follow-up and 203 CFA clearance events. For the microfilaremia clearance analysis, 129 individuals had a total of 400 person-years of follow-up with 100 microfilaremia-clearance events. The survival data concerning nontime-varying variables are summarized in Table 1.

**Table 1.** Survival Data for Time Constant Variables Used in Circulating Filarial Antigenemia and Microfilaremia Survival Models

		CFA Clearance		Microfilaremia Clearance	
Variable	Category	Person-years	No. of Events	Person-years	No. of Events
		1369	203	400	100
Sex	Male	754	112	232	61
	Female	615	91	168	39
Age at inclusion, y	5–17	304	47	87	22
	18–30	349	48	92	23
	31–45	402	59	113	24
	≥46	314	49	108	31
CFA score at inclusion	1	488	128	30	9
	2	352	51	91	28
	3	529	24	279	63
Bednets use at inclusion	No	561	82	138	33
	Yes	808	121	262	67
Fishing activities at inclusion	No	596	100	161	43
	Yes	699	95	211	52
History of sleeping outside at inclusion	No	953	159	248	63
	Yes	408	44	144	36
Village	Misay (DRC)	297	39	85	21
	Mbunkimi (DRC)	660	79	199	49
	Seke Pembe (Congo)	412	85	116	30
Study site	Bouenza (Congo)	957	118	284	70
	Kwilu (DRC)	412	85	116	30
Initial microfilarial density	0–150 Mf/mL			144	44
	151–300 Mf/mL			77	22
	>300 Mf/mL			179	34

Abbreviation: CFA, circulating filarial antigenemia; DRC, the Democratic Republic of the Congo; Mf, microfilariae.

### CFA and Microfilaremia Clearance Rates

Clearance rates with significance values are presented in Table 2. The probability of CFA clearance was negatively correlated with initial CFA score, and the probability of microfilaremia clearance was negatively correlated with initial MFD. A history of sleeping regularly in the bush decreased the probability of CFA clearance. CFA clearance was also more likely in Congo than in DRC. The probabilities for clearance of CFA for each of the 39 treatment patterns during the 5-year period are included in the Supplementary Materials 1.

**Table 2.** Univariate Clearance Rates for Circulating Filarial Antigenemia and Microfilaremia

		CFA			Microfilaremia		
Variable	Category	Clearance Rate^a^	95% CI	*P*Value^b^	Clearance Rate^a^	95% CI	*P*Value^b^
		14.8	12.9 to 17.0		25.0	20.5 to 30.4	
Sex	Male	14.8	12.0 to 18.2	.989	26.3	20.4 to 33.8	.543
	Female	14.8	12.3 to 17.9		23.2	17.0 to 31.8	
Age at inclusion, y	5–17	15.5	11.6 to 20.6	.859	25.3	16.6 to 38.4	.739
	18–30	13.7	10.4 to 18.2		25.0	16.6 to 37.6	
	31–45	14.7	11.4 to 18.9		21.2	14.2 to 31.7	
	≥46	15.6	11.8 to 20.6		28.7	20.2 to 40.8	
CFA score at inclusion	1	26.2	22.1 to 31.2	<.0001	30.0	15.6 to 57.6	.184
	2	14.5	11.0 to 19.1		30.8	21.2 to 44.6	
	3	4.5	3.0 to 6.8		22.6	17.6 to 28.9	
Bednets use at inclusion	No	14.6	11.8 to 18.1	.677	23.9	17.0 to 33.6	.752
	Yes	14.9	12.5 to 17.9		25.6	20.1 to 32.5	
Fishing activity at inclusion	No	16.8	13.8 to 20.4	.671	19.0	14.1 to 25.6	.444
	Yes	13.6	11.1 to 16.6		16.2	12.4 to 21.3	
History of sleeping outside at inclusion	No	16.7	14.3 to 19.5	.042	26.7	19.8 to 36.0	.696
	Yes	10.8	8.0 to 14.5		24.6	18.8 to 32.2	
Village	Misay (DRC)	13.1	9.6 to 18.0	<.0001	24.7	16.1 to 37.9	.859
	Mbunkimi (DRC)	12.0	9.6 to 14.9		24.6	18.6 to 32.6	
	Seke Pembe (Congo)	20.6	16.7 to 25.5		25.9	18.1 to 37.0	
Study site	Bouenza (Congo)	12.3	10.3 to 14.8	<.001	24.6	19.5 to 31.1	.826
	Kwilu (DRC)	20.6	16.7 to 25.5		25.9	18.1 to 37.0	
Initial microfilarial density	1–150 Mf/mL				30.6	22.7 to 41.0	.036
	151–300 Mf/mL				28.6	18.8 to 43.4	
	>300 Mf/mL				19.0	13.6 to 26.6	

Abbreviations: CFA, circulating filarial antigenemia; CI, confidence interval; DRC, the Democratic Republic of the Congo; Mf, microfilariae.

^a^Calculated for 100 person-years.

^b^ *P* value is calculated from significance tests using the Mantel-Haenszel method based on stratified rate ratios.

### Parametric Survival Multivariate Models for the Clearance of CFA and Microfilaremia

Results from the parametric survival model analyses are presented in Table 3. Log-logistic distribution and log-normal distribution were the best fits for time in the CFA and microfilaremia clearance models, respectively. No interactions between covariates were found. A random effect at the village level (*P* = .0277) was included in the CFA clearance model (intraclass correlation coefficient = 7.27%), but this was not significant in the microfilaremia clearance model (*P* = .346). CFA score at inclusion, frequently sleeping outdoors, and type of CFA test were all significantly associated with CFA clearance. Times to CFA clearance were significantly longer in individuals with higher initial CFA scores. Sleeping outdoors significantly increased the time to CFA clearance. Assessment of the CFA by ICT decreased the observed duration to CFA clearance. Predicted average time for clearing CFA was shorter in those who had taken 2 doses of ALB per year (3.9 years) than in those who had taken 1 or 0 dose (4.4 and 5.3 years, *P* < .001 for both comparisons). Microfilaremia clearance had a similar pattern: individuals who had taken 2 doses of ALB per year became amicrofilaremic after a mean time of 3.1 years, whereas those who had taken 1 or 0 dose per year needed 3.6 (*P* < .001) and 5.9 years (*P* < .001), respectively, to clear their microfilaremia. Time to microfilaremia clearance was also significantly longer in individuals with higher initial MFD.

**Table 3.** Results From Parametric Survival Models for Circulating Filarial Antigenemia With Village as a Random Effect and Microfilaremia Clearance

		CFA Clearance			Microfilaremia Clearance		
Variable	Category	Adjusted Time Ratio	95% CI	*P*Value	Adjusted Time Ratio	95% CI	*P*Value
Sex	Female	Ref.			Ref.		
	Male	1.01	.96 to 1.06	.718	0.94	.84 to 1.05	.281
Age, y	5–17	Ref.			Ref.		
	18–30	1.00	.93 to 1.07	.910	1.02	.87 to 1.20	.769
	31–45	1.02	.95 to 1.08	.515	1.15	.98 to 1.34	.082
	≥46	1.00	.93 to 1.07	.992	1.03	.89 to 1.20	.644
Initial CFA score	1	Ref.			Ref.		
	2	1.14	1.08 to 1.20	<.001	0.91	.74 to 1.11	.357
	3	1.40	1.31 to 1.51	<.001	1.00	.83 to 1.21	.982
Annual treatment	0 dose	1.35	1.26 to 1.45	<.001	1.82	1.46 to 2.27	<.001
	1 dose	1.12	1.06 to 1.19	<.001	1.18	1.04 to 1.34	.008
	2 doses	Ref.			Ref.		
Bednets	No	Ref.			Ref.		
	Yes	0.98	.94 to 1.03	.515	0.94	.84 to 1.04	.243
Fishing	No	Ref.			Ref.		
	Yes	0.97	.92 to 1.02	.280	1.06	.95 to 1.18	.305
Sleeping outside	No	Ref.			Ref.		
	Yes	1.09	1.03 to 1.16	.002	1.05	.94 to 1.18	.397
Test used	Filariasis Test Strip	Ref.					
	Immunochromatographic card test	0.76	.69 to .85	<.001			
Initial microfilarial density	1–150 Mf/mL				Ref.		
	151–300 Mf/mL				1.04	.92 to 1.19	.514
	>300 Mf/mL				1.28	1.14 to 1.43	<.001

Abbreviations: CFA, circulating filarial antigenemia; CI, confidence interval; Mf, microfilariae.

### Changes in MFD Over Time

**Figure 1.**
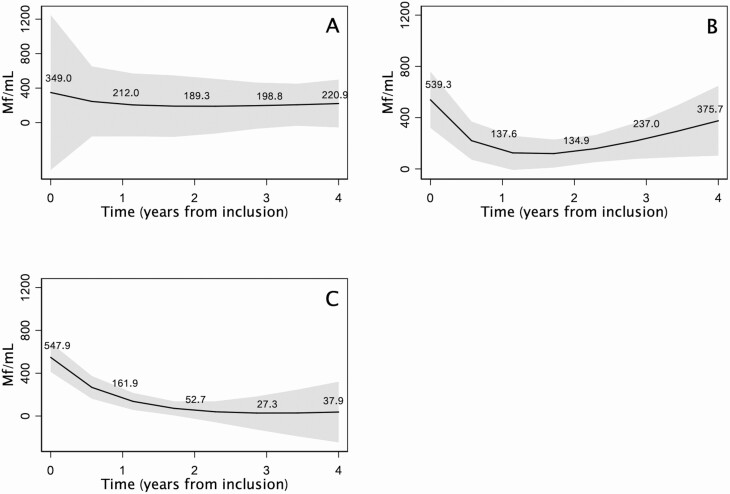
Predictions of Mf density evolution according to time (fractional polynomial of order 2) and adherence with mass drug administration (*A*, 0 dose per year; *B*, 1 dose per year; *C*, 2 doses per year). Full model and time transformations are available in Supplementary Materials 2. Abbreviation: Mf, microfilariae.

No transformation of time was required for the model. Neither the village- (*P* = .496) nor the household-level (*P* = .529) random effect was significant in the mixed model. Results from the mixed model with no random effect are presented in Table 4. MFD reduction was more rapid when individuals were adherent with MDA. The decrease in MFD was not significantly different in those who had taken 0 or 1 dose of ALB per year. Figure 1 shows predicted changes in MFD according to the number of doses taken per year with time transformation into a fractional polynomial of order 2 (see Supplementary Materials 2). All predictions were adjusted for sex, age, and initial MFD. Differences in slopes were highly significant between 0 and 2 doses of ALB (*P* = .009) and between 2 and 1 dose (*P* = .004) but not significant between 1 and 0 dose (*P* = .419).

**Table 4.** Mixed Model Results for the Evolution of Microfilariae Density

Variable	Category	Adjusted Coefficients	95% Confidence Interval	*P* Value
Sex	Female	Ref.		
	Male	−26.6	−131.2 to 77.9	.618
Age, y	5–17	Ref.		
	18–30	−93.1	−236.6 to 50.4	.203
	31–45	35.3	−103.6 to 174.1	.619
	≥46	−59.6	−196.8 to 77.5	.394
Initial circulating filarial antigenemia score	1	Ref.		
	2	−37.8	−250.9 to 175.4	.728
	3	52.6	−156.8 to 262.0	.622
Initial microfilarial density	1–200 Mf/mL	Ref.		
	> 200 Mf/mL	221.3	124.3 to 318.3	<.001
Bednets	No	Ref.		
	Yes	29.9	−61.9 to 121.8	.523
Fishing	No	Ref.		
	Yes	–18.5	−133.9 to 96.9	.753
Sleeping outside	No	Ref.		
	Yes	−21.0	−138.5 to 96.5	.753
Annual treatment	0 dose	Ref.		
	1 dose	177.8	−472.3 to 828.0	.592
	2 doses	416.6	–217.3 to 1050.5	.198
Time	Continuous	−22.0	−174.6 to 130.5	.777
Annual treatment interacted with time	0 dose	Ref.		
	1 dose	−68.6	−234.9 to 97.7	.419
	2 doses	–210.9	–369.3 to –52.44	.009

Abbreviation: Mf, microfilariae.

## Discussion

The WHO’s provisional recommendation to use semiannual MDA with ALB alone to control LF in areas where *L. loa* is coendemic was based on thin evidence. The few trials [5, 11–13] that had evaluated the effect of a single dose of ALB on LF infection had demonstrated a modest effect of the drug on MFD. In addition, 2 meta-analyses of the efficacy of a single dose of ALB alone on Mf and CFA prevalences concluded that this treatment would induce only a small to nonexistent decrease in these outcomes at 3, 6, or 12 months post-treatment [26, 27].

We conducted community trials in 2 settings to evaluate the impact of semiannual MDA with ALB on LF [18, 19]. However, a major methodological limitation of these trials was that the effect of semiannual treatment was not directly compared with that of annual treatment or no treatment. Thus, the analyses presented here provide important information regarding the added value of semiannual ALB treatment vs annual MDA or no treatment on LF infection parameters. Our longitudinal analyses from infected persons clearly demonstrate a dose-response effect for ALB treatment on CFA and on microfilaremia. Our results show that good adherence leads to faster clearance of LF infection in individuals. Both clearances were significantly associated with the number of doses of ALB taken annually and with initial infection levels. A lower initial CFA score was associated with a higher probability of CFA clearance. Therefore, the knowledge of the individual semiquantitative results at baseline may be useful to improve planning for LF elimination programs. The use of the ICT was associated with an increased probability of CFA clearance relative to use of the FTS, and this is likely due to the higher sensitivity of the FTS [28]. Although baseline CFA scores were not associated with more rapid clearance of microfilaremia, higher initial MFD increased the time required for total microfilaremia clearance.

Regarding an individual’s exposure and habits, individuals who slept regularly outdoors took longer to clear CFA. This was probably due to reinfection; prior studies have identified sleeping outdoors as a significant risk factor for LF in central Africa [21, 22]. However, the use of bednets or a history of fishing were not significantly associated with the clearances. Although the nonuse of bednets has been shown to be a risk factor for LF infection, their use in infected individuals (without MDA) was not effective for clearing infections or for reducing Mf prevalence in the time frames (3 or 4 years) of this study [29]. Data on the relationship between bednet usage and treatment adherence are included in Supplementary Materials 3. Hunting and agricultural activities were not included in the models because the numbers of hunters and farmers were small at the study sites and inclusion of these occupations would have destabilized the models. Individuals were more likely to clear CFA after MDA in Congo than in DRC. This might be due to the fact that therapeutic coverage was higher and more constant and baseline infection prevalence lower in Congo than in DRC, which may reduce transmission and, therefore, the probability that reinfection occurs.

The activity of ALB alone for LF has important implications for current protocols that rely heavily on CFA surveys in school-aged children for MDA-stopping decisions and post-MDA surveillance. Indeed, since soil-transmitted helminth programs routinely only treat children, using this demographic as a sentinel for MDA-stopping decisions may underestimate the level of community-wide transmission because LF will tend to be less prevalent in children who are treated more frequently than adults.

We elected to use parametric survival models to analyze these data because these models are more flexible and allow longitudinal analyses with time-varying variables (ie, ALB intake). In addition, they are more informative than nonparametric approaches because they provide time ratios, enable predictions of mean and median survival times, and have more power than semiparametric models. Log-logistic distribution for the CFA clearance model and log-normal distribution for the microfilaremia clearance model were the best fits for our data, and they have the advantage of not requiring proportional risk assumptions, unlike conventional Cox survival models.

The presence of bias cannot be excluded. Prevalence bias may be present. However, fewer than 11% of the population (8.5% and 10.4% for the CFA and microfilaremia clearance models, respectively) had taken ALB prior to our study. We believe that this bias, if it exists, would have had very little impact on our results. In addition, participation bias cannot be excluded: people with a high participation frequency in our study may have different characteristics than nonparticipants, including adherence with treatment.

We have mentioned that participation rates in MDA decreased over time, probably reflecting a type of fatigue on the part of some community members [19]. We believe that we have demonstrated through these new analyses that participation rates in MDA programs must be maintained at high levels to accelerate the elimination of LF in individuals and communities. Evidence from this study could be used in social mobilization programs to illustrate the importance of achieving and sustaining high rates of MDA adherence in LF elimination programs.

## Supplementary Data

Supplementary materials are available at *Clinical Infectious Diseases* online. Consisting of data provided by the authors to benefit the reader, the posted materials are not copyedited and are the sole responsibility of the authors, so questions or comments should be addressed to the corresponding author.

ciaa1232_suppl_Supplementary_Material

## Notes

***Acknowledgments.*** The authors thank the authorities and residents of the study sites for their active participation in the trials and the personnel from the ministries of health of the Congo and the Democratic Republic of the Congo for their assistance.

***Financial support***. Initial trials were funded by grant GH5342 from the Bill & Melinda Gates Foundation.

***Potential conflicts of interest.*** The authors: No reported conflicts of interest. All authors have submitted the ICMJE Form for Disclosure of Potential Conflicts of Interest.
