# Supplementary material for: Results From 2 Cohort Studies in Central Africa Show That Clearance of Wuchereria bancrofti Infection After Repeated Rounds of Mass Drug Administration With Albendazole Alone Is Closely Linked to Individual Adherence
Source: Clin Infect Dis. 2020 Aug 28;73(1):e176–83. doi: 10.1093/cid/ciaa1232 (PMC8246789; doi:10.1093/cid/ciaa1232)
Supplement: ciaa1232_suppl_Supplementary_Material [file ciaa1232_suppl_supplementary_material.docx]

| Duration of follow-up | Treatment Pattern ^a^ | N ^b^ | CFA clearance ^c^ | Proportion of CFA-negative subjects at the end of follow-up |
| --- | --- | --- | --- | --- |
| 1 year | 0 | 5 | 2 | 40.0% |
|  | 1 | 30 | 6 | 20.0% |
|  | 2 | 54 | 15 | 27.8% |
|  | **Total** | 89 | 23 | 25.8% |
| 2 years | 0 2 | 2 | 1 | 50.0% |
|  | 1 0 | 1 | 1 | 100% |
|  | 1 1 | 8 | 5 | 62.5% |
|  | 1 2 | 12 | 8 | 66.7% |
|  | 2 1 | 15 | 5 | 33.3% |
|  | 2 2 | 52 | 30 | 57.7% |
|  | **Total** | 90 | 50 | 55.6% |
| 3 years | 0 0 0 | 2 | 1 | 50.0% |
|  | 0 2 0 | 1 | 1 | 100% |
|  | 0 2 1 | 1 | 1 | 100% |
|  | 1 0 2 | 1 | 0 | 0% |
|  | 1 1 0 | 2 | 2 | 100% |
|  | 1 1 2 | 1 | 1 | 100% |
|  | 1 2 0 | 3 | 3 | 100% |
|  | 1 2 1 | 2 | 1 | 50.0% |
|  | 1 2 2 | 11 | 5 | 45.5% |
|  | 2 0 1 | 1 | 0 | 0% |
|  | 2 0 2 | 1 | 0 | 0% |
|  | 2 1 1 | 3 | 1 | 33.3% |
|  | 2 1 2 | 13 | 6 | 46.1% |
|  | 2 2 0 | 14 | 14 | 100% |
|  | 2 2 1 | 6 | 3 | 50% |
|  | 2 2 2 | 88 | 51 | 57.9% |
|  | **Total** | 150 | 90 | 60.0% |
| 4 years | 0 1 2 0 | 1 | 1 | 100% |
|  | 0 2 1 2 | 1 | 1 | 100% |
|  | 1 1 2 0 | 1 | 1 | 100% |
|  | 1 1 2 2 | 1 | 0 | 0% |
|  | 1 2 0 0 | 1 | 1 | 100% |
|  | 1 2 2 2 | 6 | 2 | 33.3% |
|  | 2 0 2 1 | 1 | 0 | 0% |
|  | 2 1 2 0 | 1 | 1 | 100% |
|  | 2 1 2 2 | 1 | 1 | 100% |
|  | 2 2 0 0 | 4 | 4 | 100% |
|  | 2 2 1 0 | 2 | 2 | 100% |
|  | 2 2 2 0 | 8 | 8 | 100% |
|  | 2 2 2 1 | 3 | 2 | 66.7% |
|  | 2 2 2 2 | 34 | 16 | 47.0% |
|  | **Total** | 65 | 40 | 61.5% |
| **Total** | | 394 | 203 | 51.5% |

**Supplementary material 1**. Proportion of subjects who were CFA-negative at the end of their follow-up according to the number of albendazole doses received during each year of follow-up ("treatment pattern")

^a^ the first digit corresponds to the number of treatment received during the first year of follow-up, the second digit corresponds to the number of treatment received during the second year of follow-up, etc.

^b^ numbers of subjects corresponding to each pattern

^c^ numbers of subjects who experienced CFA negativation during the last year of follow-up.

| **Variables** | **Categories** | **Adjusted coefficients** | **95% CI ^a^** | ***P*** |
| --- | --- | --- | --- | --- |
| Sex | Female | Ref. |  |  |
|  | Male | -47.1 | -147.5 – 53.3 | .358 |
| Age | 5 – 17 years | Ref. |  |  |
|  | 18 – 30 years | -68.5 | -206.4 – 69.5 | .331 |
|  | 31 – 45 years | 28.6 | -103.7 – 161.0 | .672 |
|  | ≥ 46 years | -60.4 | -191.8 – 70.9 | . 367 |
| Initial CFA score | 1 | Ref. |  |  |
|  | 2 | -43.7 | -247.2 – 159.8 | .674 |
|  | 3 | 56.1 | -145.0 – 257.3 | .584 |
| Initial MFD | 1 – 200 Mf/mL | Ref. |  |  |
|  | > 200 Mf/mL | 175.8 | 81.6 – 270.0 | < .001 |
| Bednets | No | Ref. |  |  |
|  | Yes | -11.0 | -100.9 – 78.9 | .810 |
| Fishing | No | Ref. |  |  |
|  | Yes | 16.3 | -94.3 – 126.9 | .773 |
| Sleep outside | No | Ref. |  |  |
|  | Yes | -31.7 | -143.2 – 79.7 | .577 |
| Annual treatment | 0 dose | Ref. |  |  |
|  | 1 dose | -78.7 | -455.4 – 297.9 | .682 |
|  | 2 doses | -62.5 | -427.3 – 302.3 | .737 |
| FP1 (Time)^b^ | Continuous | -286.9 | -2221.3 – 1647.5 | .771 |
| FP2 (Time)^c^ | Continuous | 128.8 | -806.3 – 1063.9 | .787 |
| Annual treatment interacted with FP1 (Time) | 0 dose | Ref. |  |  |
|  | 1 dose | -654.1 | -2698.4 – 1390.3 | .531 |
|  | 2 doses | -450.9 | -2417.8 – 1515.9 | .653 |
| Annual treatment interacted with FP2 (Time) | 0 dose | Ref. |  |  |
|  | 1 dose | 392.7 | -631.7 – 1417.1 | .452 |
|  | 2 doses | 132.6 | -841.5 – 1106.7 | .790 |

**Supplementary material 2. Model results for the evolution of Mf density (MFD) with time as a fractional polynomial of order 2.**

^a^ 95% confidence intervals

^b^ the transformation for the Fractional polynomial 1 is: log(time) - .7406723224

^c^ the transformation for the Fractional polynomial 2 is: log(time)^2^ - .5485954892

|  | **Treatment adherence N (%)** | | | **Total** |
| --- | --- | --- | --- | --- |
| **Bed net usage** | **0 dose in the year** | **1 dose in the year** | **2 doses in the year** |  |
| **No** | 60 (55.6%) | 109 (37.5%) | 253 (30.8%) | 422 (34.6%) |
| **Yes** | 48 (44.4%) | 182 (62.5%) | 569 (69.2%) | 799 (65.4%) |
| **Total** | 108 | 291 | 822 | 1221 |

Cuzick test: Z = 4.99*2, P* = 0.0001

Spearman coefficient between bed net usage (yes/no) and treatment adherence (0, 1 or 2 doses) for all observations: 0.129 (*P* < 0.0001)

**Supplementary material 3**. Relationship between bed nets use and treatment adherence for all observations included in parametric survival model on CFA clearance (N: number of observations, %: percentage of observations according to bed net usage)
